# Guzhi Zengsheng Zhitongwan, a Traditional Chinese Medicinal Formulation, Stimulates Chondrocyte Proliferation through Control of Multiple Genes Involved in Chondrocyte Proliferation and Differentiation

**DOI:** 10.1155/2018/7265939

**Published:** 2018-09-12

**Authors:** Baojin Yao, Bocheng Lu, Hongwei Gao, Mei Zhang, Xiangyang Leng, Daqing Zhao

**Affiliations:** ^1^Jilin Ginseng Academy, Changchun University of Chinese Medicine, Changchun, Jilin 130117, China; ^2^The Affiliated Hospital of Changchun University of Chinese Medicine, Changchun, Jilin 130117, China; ^3^Innovation Practice Center, Changchun University of Chinese Medicine, Changchun, Jilin 130117, China

## Abstract

Chinese materia medica (CMM) are essential components of traditional Chinese medicine, and Chinese medicinal formulas consisting of 2 or more types of CMM are widely used. These formulations have played a pivotal role in health protection and disease control for thousands of years. Guzhi Zengsheng Zhitongwan (GZZSZTW), which represents one of the Chinese medicinal formulations, has been used for several decades to treat joint diseases. However, the exact molecular mechanism underlying its efficacy in treating osteoarthritis remains to be elucidated. In the present study, we investigated the effects of GZZSZTW on primary chondrocytes. We demonstrated that GZZSZTW significantly promoted chondrocyte viability, maintained chondrocytes in a continuous proliferative state, and prevented their further differentiation. These effects were achieved by the synergistic interactions of various herbs and their active components in GZZSZTW through an increase in the expression levels of functional genes participating in chondrocyte commitment and proliferation and a decrease in the expression levels of genes involved in chondrocyte differentiation. GZZSZTW treatment also decreased the expression levels of genes that inhibited chondrocyte proliferation. Thus, this study has greatly deepened the current knowledge about the molecular effects of GZZSZTW on chondrocytes. It has also shed new light on possible strategies to further prevent and treat cartilage-related diseases by using traditional Chinese medicinal formulations.

## 1. Introduction

It is well-known that Youyou Tu won the 2015 Nobel Prize in Physiology or Medicine for her discoveries concerning a novel therapy for malaria using artemisinin, which is derived from a traditional herbal medicine called qinghaosu. Since then, Chinese materia medica (CMM) has attracted worldwide attention and has extended to the forefront of the global research community [1].

CMM is an essential component of traditional Chinese medicine; it relies on Chinese medicinal formulas consisting of 2 or more types of CMM and has played a pivotal role in health protection and disease control for thousands of years [2]. In other words, CMM have already provided outstanding clinical results, since they have been tested in human experiments with long-term observation periods and in clinical trials [3]. For instance, Guzhi Zengsheng Zhitongwan (GZZSZTW), a Chinese medicinal formulation created by the national medical master professor Bailing Liu, has been used in the Affiliated Hospital of Changchun University of Chinese Medicine for several decades to treat joint diseases such as osteoarthritis. Clinically, this formula is routinely taken in the form of orally administered pills, which are only produced in the hospital's internal preparation center to treat patients, and it is not yet available commercially. Although it is effective and widely used in the treatment of joint diseases in our hospital, the precise molecular mechanism underlying its efficacy in treating osteoarthritis remains to be elucidated.

Osteoarthritis (OA) is the most common degenerative joint and cartilage disease and is associated with pain and disability. However, no suitable treatment for OA exists, because of the poor self-healing capacity of damaged cartilage [4]. Chondrocytes are the only cells residing in cartilage that control cartilage structure and function by regulating extracellular matrix turnover and maintaining cartilage homeostasis [5]. Thus, investigating the effect of the Chinese medicinal formulation GZZSZTW on chondrocytes is therefore very important for further dissecting the molecular mechanism behind its successful clinical treatment of osteoarthritis.

In the present study, we prepared aqueous extracts of GZZSZTW. We isolated primary chondrocytes from neonatal mouse rib cartilage and investigated the effects of GZZSZTW on the proliferation of chondrocytes. We further performed RNA-seq to analyze the gene expression pattern of chondrocytes in response to treatment with GZZSZTW. We demonstrated that GZZSZTW significantly promoted chondrocyte viability, maintained chondrocytes in a continuous proliferative state, and suppressed their further differentiation by increasing the expression levels of genes that promote chondrocyte commitment and proliferation and a decrease in the expression levels of genes that inhibit chondrocyte proliferation and promote chondrocyte differentiation.

## 2. Materials and Methods

### 2.1. Preparation of the GZZSZTW Aqueous Extract

GZZSZTW was obtained from the Affiliated Hospital of Changchun University of Chinese Medicine (Changchun, China). The formulation of GZZSZTW consisted of 7 types of CMM, namely,* Rehmannia glutinosa *(Gaertn.) DC.,* Spatholobus suberectus *Dunn,* Epimedium brevicornu Maxim *(K.S.Hao)*, Raphanus sativus L. *(Hook. f. & T. Anderson) (baked),* Drynaria fortunei (*Kunze ex Mett.) J.Sm. (baked),* Cynomorium coccineum subsp. songaricum (Rupr.) *(J.Léonard), and* Cibotium barometz (L.) *(J.Sm). The CMM mixture of GZZSZTW was extracted with distilled water by a reflux method and was then filtered through a 0.45-*μ*m Hollow Fiber Cartridge (GE Healthcare, USA). The filtrate was freeze-dried by a Heto PowerDry LL3000 Freeze Dryer (Thermo, USA) and stored at -80°C.

### 2.2. Isolation of Primary Chondrocytes

Animal experiments were approved by the Ethical Committee for Animal Research of Changchun University of Chinese Medicine. Primary chondrocytes from the rib cartilage of neonatal mice were isolated as previously described [6]. Briefly, cartilage from the rib cages of neonatal C57BL/6J mice was digested for 45 minutes with 3 mg/ml collagenase D and then overnight with 0.5 mg/ml collagenase D (Sigma, USA). The cells were centrifuged and resuspended in DMEM/F12 medium (Thermo, USA) containing 5% FCS (Thermo, USA) and 1% penicillin/streptomycin (Sigma, USA).

### 2.3. Cell Proliferation Assay

The Cell Counting Kit-8 (CCK-8) (Sigma, USA) was used to evaluate chondrocyte proliferation according to the manufacturer's protocol. Briefly, primary chondrocytes were seeded into 96-well cell culture plates at a density of 5 × 10^4^ cells/ml (100 *μ*l per well) and cultured at 37°C in a 5% CO_2_ incubator (Thermo, USA) for 4 h. The cell culture medium was then discarded, and each well was rinsed thoroughly with fresh culture medium. The chondrocytes were treated with GZZSZTW at different concentrations (0, 0.2, 0. 4, 0.6, 0.8, and 1.0 mg/ml dissolved in culture medium; 100 *μ*l per well) and subsequently cultured for 24 h. Deer antler extract (DAE) was used as a reference drug and added to the cells in the same way as GZZSZTW. Next, 10 *μ*l of CCK-8 reagent was added, and the cells were incubated for 1 h. The absorbance was measured at 450 nm using an Infinite 200 PRO plate reader (Life Sciences, USA). The cell proliferation rate was calculated as the percentage of cell viability after treatment with GZZSZTW and DAE at different concentrations, respectively.

### 2.4. RNA Purification and Illumina Sequencing

Primary chondrocytes were isolated as described above, seeded into 6-well cell culture plates at a density of 1 × 10^5^ cells/well, and incubated for 4 h. Chondrocytes were treated with GZZSZTW for 24 h or left untreated. Then, the cell culture medium was discarded, and each well was rinsed thoroughly with cold PBS buffer. Total RNA was isolated using TRIzol (Invitrogen, USA) in accordance with the manufacturer's protocol. RNA integrity was evaluated using the Agilent 2100 Bioanalyzer (Agilent Technologies, USA). Paired-end mRNA libraries were generated using the TruSeq Stranded mRNA kit (Illumina, USA) according to the manufacturer's protocol. High-throughput sequencing of the mRNA libraries was performed on an Illumina HiSeq 2500 platform (Illumina, USA).

### 2.5. RNA-Seq Data Analysis

After RNA-seq, clean reads were obtained by trimming the raw reads to remove the low-quality reads and adapter sequences. The data sets were deposited in the NCBI Sequence Read Archive (SRA) database under accession number SRP125978. The clean reads were aligned with the mouse (*Mus musculus*) reference genome using HISAT [7]. The FPKM algorithm was used to measure the gene expression levels [8]. BLAST was used to perform annotations against the nonredundant (NR) and Swiss-Prot protein databases. DEGseq was used to analyze the differentially expressed genes [9]. Genes with a log_2_ fold change ≥ 1 or ≤ -1 and with a p value ≤ 0.001 were considered to be differentially expressed.

### 2.6. Quantitative Real-Time PCR (qRT-PCR) Verification

The expression levels of differentially expressed genes were validated by qRT-PCR. Briefly, total RNA was isolated using TRIzol (Invitrogen, USA) in accordance with the manufacturer's protocol. cDNA was synthesized using the iScript cDNA Synthesis Kit (Bio-Rad, USA) and amplified using SsoAdvanced Universal SYBR® Green Supermix (Bio-Rad, USA) on a CFX Connect Real-Time PCR Detection System (Bio-Rad, USA) under standard amplification conditions. The gene expression levels were normalized to the mouse beta-actin gene (*Actb*) and calculated using the 2^−ΔΔCT^ method [10].

## 3. Results

### 3.1. GZZSZTW Promotes Proliferation in Primary Chondrocytes in a Dose-Dependent Manner

**Figure 1:**
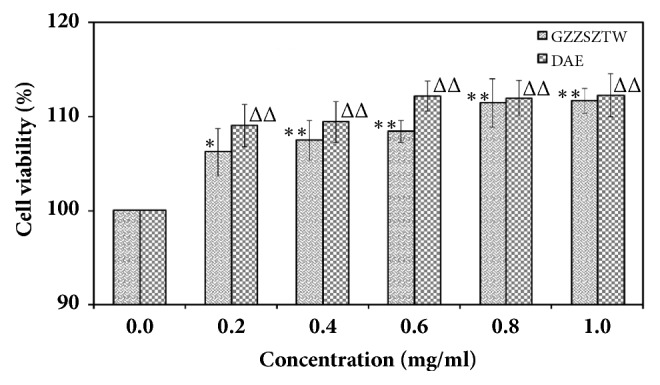
Cell viability assay in primary chondrocytes treated with GZZSZTW and DAE. The CCK-8 kit was used to detect chondrocyte proliferation following treatment with GZZSZTW and DAE at increasing concentrations (0, 0.2, 0.4, 0.6, 0.8, and 1.0 mg/ml) for 24 h, respectively. Cell viabilities were normalized and calculated relative to the untreated group (0 mg/ml). Data are presented as the mean and standard deviation for technical triplicates in an experiment representative of several other independent experiments. *∗* (Δ) represents p <0.01 and *∗∗* (ΔΔ) represents p <0.001, computed by the t-test for the difference in cell viability in response to GZZSZTW and DAE treatment, respectively.

The effect of GZZSZTW on chondrocyte proliferation was measured by the CCK-8 assay. Deer antler extract (DAE), which has been shown to promote chondrocyte viability and keep chondrocytes proliferating continuously, while blocking maturation and further differentiation in a dose-dependent manner, was used as a reference drug [11]. As shown in Figure 1, chondrocyte viability was significantly increased in a dose-dependent manner with GZZSZTW treatment compared with the untreated control group (0 mg/ml). Since treatment with GZZSZTW at the concentrations of 0.8 mg/ml and 1.0 mg/ml had a similar effect on chondrocyte viability, a concentration of 0.8 mg/ml GZZSZTW was selected for use in the following experiments.

### 3.2. Sequencing, Genome Mapping, and Functional Annotation

After Illumina sequencing and data processing, 40,958,510 and 40,961,200 clean reads were obtained from primary chondrocytes not treated with GZZSZTW (Blank) and those treated with GZZSZTW, respectively, as shown in Table 1. The quality assessment showed that the Q30 percentages were greater than 94%, and the GC content percentages were approximately 52%. For the Blank and GZZSZTW-treated samples, 36,258,888 and 36,392,470 reads were aligned to the mouse genome, respectively. In total, 15,788 out of 15,908 (Blank) and 15,566 out of 15,676 (GZZSZTW) transcripts were annotated against the nonredundant (NR) NCBI protein database and Swiss-Prot database, respectively.

### 3.3. Comparative Analysis of Differentially Expressed Genes

The differential expression analysis identified 229 genes that were significantly differentially expressed between the GZZSZTW-treated and Blank groups (log_2_ fold change ≥ 1 or ≤ -1 and p ≤0.001), including 139 upregulated genes and 90 downregulated genes (GZZSZTW versus Blank), as shown in Table 2.

### 3.4. GZZSZTW Increases the Expression Levels of Multiple Genes That Positively Regulate Chondrocyte Proliferation

Because GZZSZTW was able to promote proliferation in primary chondrocytes, we first analyzed the differentially expressed genes that positively regulated chondrocyte proliferation. Based on the results from the RNA-seq analysis, we identified 10 differentially expressed genes that directly promoted cell proliferation, including Tnfaip2, Chi3l1, Tnf, Pfkfb3, Sox8, Jag1, Mafb, Pla2g7, Hnrnpa1, and E2f3. The expression levels of these genes were significantly increased in response to GZZSZTW treatment, as shown in Table 3.

### 3.5. GZZSZTW Treatment Maintains Chondrocyte Proliferation by Decreasing the Expression Levels of Inhibitors of Cell Proliferation

We then analyzed the differentially expressed genes that participated in the inhibition of cell proliferation. In total, the expression levels of 7 genes were significantly decreased in response to GZZSZTW treatment. These genes are typically downregulated when cells are in a proliferative state and play a crucial role in inhibiting cell proliferation, as shown in Table 4.

### 3.6. GZZSZTW Treatment Maintains Chondrocyte Proliferation by Promoting Chondrocyte Proliferation and Suppressing Chondrocyte Differentiation

According to the RNA-seq analysis, the expression levels of pancartilaginous early chondrocyte markers including Sox9, Sox5, Sox6, Acan, Col2a1, Col9a1, Col11a1, Hapln1, and Wwp2 were slightly increased in response to GZZSZTW treatment, as shown in Table 5.

Furthermore, the expression levels of proliferating and prehypertrophic chondrocyte markers including Fgfr3, Matn1, Comp, Ptch1, Runx2, and Runx3 were also slightly increased in response to GZZSZTW treatment. However, the expression levels of the major prehypertrophic and hypertrophic chondrocyte markers including Pth1r, Sp7, Ihh, Bmp6, and Ibsp either decreased slightly or exhibited almost no change in response to GZZSZTW treatment, as shown in Table 6.

### 3.7. Validation of RNA-Seq Data by qRT-PCR

To validate the accuracy of the RNA-seq results, we selected 8 of the differentially expressed genes (Tnfaip2, Chi3l1, Sox8, Jag1, Rhob, Dusp6, Rad9a, and Filip1l) and verified their expression profiles using qRT-PCR. The specific primers used in this experiment are listed in Table 7.

**Figure 2:**
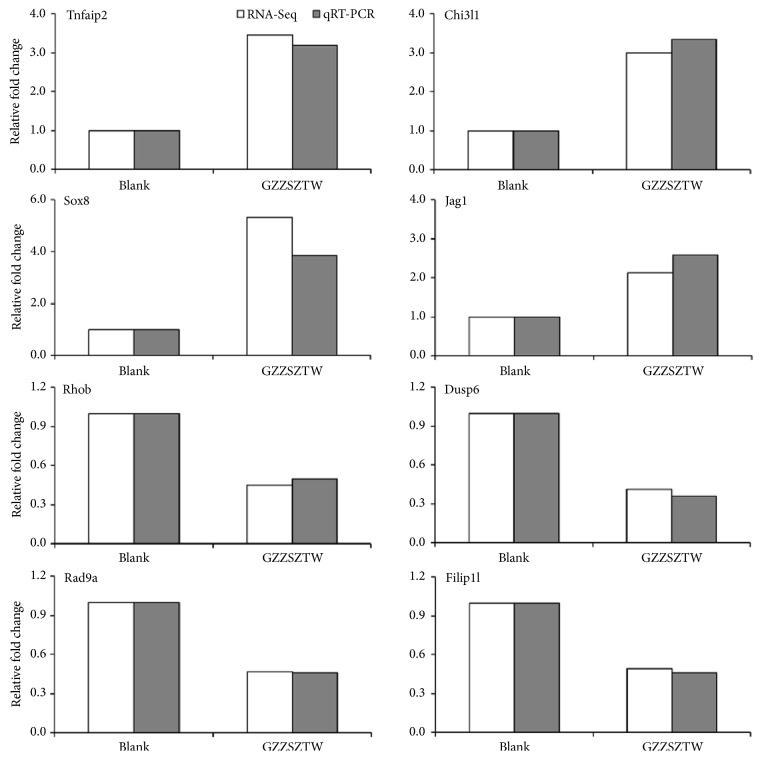
Gene expression levels of differentially expressed genes validated by qRT-PCR. Gene expression levels for individual genes are presented as the fold change between the GZZSZTW-treated group and the untreated control group.

The relative fold change of each gene was normalized to the internal reference gene* Actb*. The expression levels of the selected differentially expressed genes measured by qRT-PCR were consistent with the results of the RNA-seq analysis, as shown in Figure 2.

## 4. Discussion

GZZSZTW, a Chinese medicinal formulation widely used for treating joint diseases, has been used in the Affiliated Hospital of Changchun University of Chinese Medicine for several decades. However, the precise molecular mechanism underlying the ability of GZZSZTW to treat these diseases remains to be elucidated. In the present study, we investigated the effects of GZZSZTW on primary mouse chondrocytes using state-of-the-art RNA-seq technology. According to the results from the CCK-8 assay, GZZSZTW significantly promoted proliferation in primary chondrocytes in a dose-dependent manner. Furthermore, GZZSZTW almost showed the same effect as DAE at the concentrations of 0.8 mg/ml and 1.0 mg/ml. We then performed RNA-seq to further explore the mechanisms responsible for regulating cell proliferation in response to GZZSZTW treatment.

We first analyzed the differentially expressed genes involved in enhancing cell proliferation. Our results indicated that GZZSZTW significantly increased the expression levels of multiple genes involved in promoting cell proliferation, most of which are highly expressed in tumor cells, such as Tnfaip2, Chi3l1, Tnf, Pfkfb3, Sox8, Jag1, Mafb, Pla2g7, Hnrnpa1, and E2f3. Expression of the Tnfaip2 gene is induced in response to Tnf, which plays a pivotal role in tumor formation and growth. Overexpression of Tnfaip2 significantly promotes tumor cell proliferation, and silencing of Tnfaip2 suppresses proliferation [12]. Chi3l1 is highly expressed in various tumors and possesses oncogenic properties. Chi311 promotes cell proliferation in a similar manner to insulin-like growth factor 1 [13]. Pfkfb3 is overexpressed in many cancers, and it promotes cell proliferation through accelerating cell cycle progression and suppressing apoptosis [14]. Sox8 is highly expressed in many tumor cells, and downregulation of Sox8 suppresses tumor cell proliferation [15]. Jag1, a ligand for the Notch family of receptors, is highly expressed in a variety of tumors. Overexpression of Jag1 enhances tumor cell proliferation [16]. Mafb is a member of the Maf family of transcription factors. Overexpression of Mafb enhances tumor cell proliferation, whereas its knockdown inhibits tumor cell proliferation [17]. Pla2g7 is a cancer-selective biomarker; silencing of Pla2g7 is an antiproliferative and proapoptotic therapeutic approach in cancer treatment [18]. Hnrnpa1 is a member of the heterogeneous nuclear ribonucleoprotein family and is highly expressed in growing mammalian cells. Knockdown of Hnrnpa1 inhibits tumor cell proliferation [19]. E2f3, a member of the E2F family of transcription factors, is critical for the transcriptional activation of genes that control proliferation in both normal and tumor cells [20]. Our results indicated that GZZSZTW treatment significantly promoted chondrocyte proliferation by upregulating the expression levels of functional genes involved in the promotion of cell proliferation.

Consistent with the results discussed above, the expression levels of several functional genes involved in the inhibition of cell proliferation were significantly decreased in response to GZZSZTW treatment, most of which act as tumor suppressors, including Rhob, Dusp6, Plk3, Fgf21, Rad9a, Filip1l, and Rasl11b. Rhob is a member of the Ras homolog family. Rhob serves as a tumor suppressor, and loss of Rhob expression has been reported in a variety of tumor cells [21]. Dusp6 is a negative regulator of tumor cell proliferation, and its expression is significantly decreased in many types of invasive tumor cells. Overexpression of Dusp6 suppresses tumor cell proliferation [22]. Plk3 is a member of the Plk family. Plk3 is downregulated in various types of tumor cells and inhibits tumor cell proliferation and tumorigenesis [23]. Fgf21 is an endocrine factor that inhibits chondrocyte proliferation and thus reduces skeletal growth [24]. Rad9a is a cell cycle checkpoint control protein and is required for cell cycle arrest and DNA damage repair in response to DNA damage [25]. Filip1l is downregulated in many types of tumor cells, and its overexpression results in inhibition of cell proliferation and increased apoptosis [26]. Rasl11b is a member of the Ras-like protein family, and its overexpression leads to decreased cell proliferation [27]. Our results indicated that GZZSZTW treatment significantly promoted chondrocyte proliferation by downregulating the expression levels of functional genes involved in the inhibition of cell proliferation.

According to our RNA-seq analysis, the expression levels of the functional genes involved in chondrocyte proliferation and differentiation were also changed slightly in response to GZZSZTW treatment. For instance, the expression levels of pancartilaginous early chondrocyte markers, including Sox9, Sox5, Sox6, Acan, Col2a1, Col9a1, Col11a1, Hapln1, and Wwp2, were slightly increased in response to GZZSZTW treatment. Sox9 is a master transcription factor that plays a major role during cartilage development through regulation of its target genes, including Sox5, Sox6, Acan, Col2a1, Col9a1, Col11a1, Hapln1, and Wwp2 [28–30]. We further investigated the expression levels of growth plate cartilage markers at different stages of differentiation. The expression levels of markers of proliferating and prehypertrophic chondrocytes, including Fgfr3, Matn1, Comp, Ptch1, Runx2, and Runx3, were slightly increased in response to GZZSZTW treatment. However, the expression levels of the major markers of prehypertrophic and hypertrophic chondrocytes, including Pth1r, Sp7, Ihh, Bmp6, and Ibsp, either decreased slightly or exhibited almost no change in response to GZZSZTW treatment. These results indicated that GZZSZTW treatment facilitated chondrogenesis, maintained chondrocytes in a proliferative state, and prevented their further differentiation into hypertrophic chondrocytes. However, we also observed that the expression level of Col10a1, a specific marker of prehypertrophic and hypertrophic chondrocytes in growth plate cartilage, was slightly increased in response to GZZSZTW treatment. This observation might be explained by the regulatory function of Sox9, which also serves as an upstream regulator of Col10a1 during the progression toward the hypertrophic stage. Sox9 sustains proliferation in columnar chondrocytes and is also required for chondrocyte hypertrophy, a stage that also includes Col10a1 expression [31].

GZZSZTW is designed according to professor Bailing Liu's clinical experiences under the guidance of fundamental theories of traditional Chinese medicine. Among the seven types of CMM in GZZSZTW,* Rehmannia glutinosa and Cynomorium coccineum subsp. songaricum (Rupr.) *are important CMM with the ability to nourish the “kidney”. In tradition Chinese medicine, kidney is never a definition in anatomic sense but a functional system which plays crucial roles in storing essence and regulating growth, development, and reproduction [32, 33].* Epimedium brevicornu Maxim, *a traditional Chinese herbal medicine, has been widely used in China for the treatment of various diseases, such as arthritis and osteoporosis [34]. Icariin, which is considered the major bioactive component of this herbal medicine, has been shown to suppress articular cartilage and bone loss and prevents joint destruction [35]. The traditional Chinese herbal medicine* Drynaria fortunei* is commonly used to treat musculoskeletal traumatic disorders, such as bone fracture and osteoarthritis [36, 37]. Propinqualin, which is similar in chemical structure to the natural phytoestrogens naringenin and genistein, has been considered as the putative active ingredient of* Drynaria fortunei* [38]. However, the role of propinqualin in the regulation of chondrocyte is still largely unknown.* Cibotium barometz *is widely used in Chinese medicinal formulations for the clinical treatment of osteoarthritis. Polysaccharides, one of the most important bioactive components extracted from* Cibotium barometz*, have been shown to stimulate chondrocyte proliferation in vitro by promoting G1/S cell cycle transition [39].

*Spatholobus suberectus* Dunn and* Raphanus sativus L. *are two types of medicinal and edible plants in traditional Chinese medicine.* Spatholobus suberectus* Dunn has been widely used as dietary supplements in addition to its traditional prescription for treating anemia, arthralgia, inflammation, and arthritis. It has been shown that gallic acid is the major compound with the anti-inflammatory effects in* Spatholobus suberectus Dunn* [40]. Im and colleagues have shown that* Spatholobus suberectus* Dunn has a potential therapeutic effect for treating osteoarthritis through preventing extracellular matrix destruction in articular cartilage [41].* Raphanus sativus L.* is an edible root vegetable that is commonly used around the world. The seeds of* Raphanus sativus L *have long been used as anti-inflammatory traditional herbal medicine, and it has been shown that sinapic acid is the major active constituent [42]. Taken together, our results indicated that various herbs and their active components in GZZSZTW synergistically promoted chondrocyte viability and proliferation, but inhibited chondrocyte differentiation, extracellular matrix destruction, and inflammation.

## 5. Conclusions

The present study demonstrated that the Chinese medicinal formulation GZZSZTW, which has been used for several decades to treat joint diseases (e.g., osteoarthritis), significantly promoted chondrocyte viability and proliferation, maintained chondrocytes in a continuous proliferative state, and inhibited further differentiation. These effects were achieved by the synergistic interactions of various herbs and their active components in GZZSZTW, which increased the expression levels of functional genes participating in chondrocyte commitment and proliferation and decreasing the expression levels of genes involved in chondrocyte differentiation. GZZSZTW treatment also decreased the expression levels of genes that inhibit chondrocyte proliferation. Thus, this study has greatly deepened the current knowledge about the molecular effects of GZZSZTW on chondrocytes. This study has also shed new light on possible strategies to further prevent and treat cartilage-related diseases by using traditional Chinese medicinal formulations.

## Figures and Tables

**Table 1:** Statistics for the sequencing and assembly results.

Statistics	Blank	GZZSZTW
Clean reads	40,958,510	40,961,200
Q30 percentage	94.38	94.42
GC percentage	52.31	52.49
Total mapped reads	36,258,888	36,392,470
Total transcripts	15,908	15,676
Known transcripts	15,788	15,566

**Table 2:** Statistical analysis of differentially expressed genes (GZZSZTW versus Blank).

Statistics	Number
Differentially expressed mRNAs	229
Upregulated mRNAs	139
Downregulated mRNAs	90

**Table 3:** Differentially expressed genes that positively regulate chondrocyte proliferation.

Gene name	Blank (FPKM)	GZZSZTW (FPKM)	log_2_ fold change (GZZSZTW /Blank)	p value
Tumor necrosis factor alpha-induced protein 2 (Tnfaip2)	37.69	129.86	1.78	0
Chitinase-3-like protein 1 (Chi3l1)	40.76	122.14	1.58	1.55E-121
Tumor necrosis factor (Tnf)	9.18	24.24	1.40	7.31E-21
6-phosphofructo-2-kinase/fructose-2,6-bisphosphatase 3 (Pfkfb3)	9.68	22.87	1.24	1.76E-51
Transcription factor SOX-8 (Sox8)	2.99	15.93	2.41	1.25E-50
Protein jagged-1 (Jag1)	6.29	13.38	1.09	3.80E-27
Transcription factor MafB (Mafb)	5.08	10.76	1.08	6.05E-14
Platelet-activating factor acetylhydrolase (Pla2g7)	4.33	9.88	1.19	1.02E-08
Heterogeneous nuclear ribonucleoprotein A1 (Hnrnpa1)	0.01	6.60	9.37	1.87E-21
Transcription factor E2F3 (E2f3)	2.47	5.44	1.14	2.88E-11

**Table 4:** Differentially expressed genes that negatively regulate chondrocyte proliferation.

Gene name	Blank (FPKM)	GZZSZTW (FPKM)	log_2_ fold change (GZZSZTW /Blank)	p value
Rho-related GTP-binding protein RhoB (Rhob)	118.64	53.33	-1.15	1.51E-97
Dual specificity protein phosphatase 6 (Dusp6)	18.05	7.41	-1.28	1.37E-23
Serine/threonine-protein kinase PLK3 (Plk3)	16.41	6.27	-1.39	3.09E-19
Fibroblast growth factor 21 (Fgf21)	14.80	7.37	-1.01	7.27E-05
Cell cycle checkpoint control protein RAD9A (Rad9a)	13.54	6.37	-1.09	1.81E-10
Filamin A-interacting protein 1-like (Filip1l)	9.78	4.84	-1.01	4.77E-11
Ras-like protein family member 11B (Rasl11b)	8.48	3.96	-1.10	1.45E-06

**Table 5:** Gene expression levels of pancartilaginous early chondrocyte markers.

Gene name	Blank (FPKM)	GZZSZTW (FPKM)	log_2_ fold change (GZZSZTW /Blank)	p value
Transcription factor SOX-9 (Sox9)	22.35	28.25	0.34	1.39E-06
Transcription factor SOX-5 (Sox5)	2.53	5.17	1.03	7.95E-14
Transcription factor SOX-6 (Sox6)	11.16	13.09	0.23	1.24E-10
Aggrecan core protein (Acan)	439.94	554.62	0.33	7.55E-177
Collagen alpha-1(II) chain (Col2a1)	16294.32	16870.22	0.05	3.43E-96
Collagen alpha-1(IX) chain(Col9a1)	1024.49	1188.74	0.21	4.10E-92
Collagen alpha-1(XI) chain (Col11a1)	838.24	1020.09	0.28	1.47E-232
Hyaluronan and proteoglycan link protein 1 (Hapln1)	117.28	132.39	0.17	5.60E-10
NEDD4-like E3 ubiquitin-protein ligase WWP2 (Wwp2)	140.53	166.42	0.24	2.99E-14

**Table 6:** Gene expression levels of growth plate chondrocyte markers.

Gene name	Blank (FPKM)	GZZSZTW (FPKM)	log_2_ fold change (GZZSZTW /Blank)	p value
Fibroblast growth factor receptor 3 (Fgfr3)	57.13	97.35	0.77	2.34E-80
Cartilage matrix protein (Matn1)	102.96	124.45	0.27	1.06E-08
Cartilage oligomeric matrix protein (Comp)	712.93	775.11	0.12	1.77E-13
Protein patched homolog 1 (Ptch1)	3.92	5.44	0.47	2.92E-03
Runt-related transcription factor 2 (Runx2)	7.43	10.01	0.43	3.02E-05
Runt-related transcription factor 3 (Runx3)	4.68	6.26	0.42	7.23E-03
Parathyroid hormone/parathyroid hormone-related peptide receptor (Pth1r)	55.23	54.53	-0.02	8.96E-01
Transcription factor Sp7 (Sp7)	3.76	4.04	0.10	4.23E-01
Indian hedgehog protein (Ihh)	0.57	0.51	-0.16	4.99E-01
Collagen alpha-1(X) (Col10a1)	92.05	122.37	0.41	1.71E-25
Bone morphogenetic protein 6 (Bmp6)	4.38	5.37	0.29	1.19E-01
Bone sialoprotein 2 (Ibsp)	78.3	72.73	-0.11	1.04E-01

**Table 7:** Primer sequences used in qRT-PCR validation.

Gene	Primer	Sequence
Tnfaip2	Forward primer	AGGAGGAGTCTGCGAAGAAGA
	Reverse primer	GGCAGTGGACCATCTAACTCG
Chi3l1	Forward primer	GTACAAGCTGGTCTGCTACTTC
	Reverse primer	ATGTGCTAAGCATGTTGTCGC
Sox8	Forward primer	CGAGGGGATACTGCTGAGG
	Reverse primer	AGCTCTGCGTTATGGAGATGC
Jag1	Forward primer	CCTCGGGTCAGTTTGAGCTG
	Reverse primer	CCTTGAGGCACACTTTGAAGTA
Rhob	Forward primer	GTGCCTGCTGATCGTGTTCA
	Reverse primer	CCGAGAAGCACATAAGGATGAC
Dusp6	Forward primer	ATAGATACGCTCAGACCCGTG
	Reverse primer	ATCAGCAGAAGCCGTTCGTT
Rad9a	Forward primer	GGCTGTCCATTCGCTATCCC
	Reverse primer	GTGGGGCAAAAAGGAAGCAG
Filip1l	Forward primer	AGCACTCAGTCGGCAAATTGA
	Reverse primer	AGCCTCTTATTGAGGTCTCTGC
Actb	Forward primer	ACCTTCTACAATGAGCTGCG
	Reverse primer	CTGGATGGCTACGTACATGG

## Data Availability

The data sets were deposited in the NCBI Sequence Read Archive (SRA) database under accession number SRP125978.
